# Hypoxic preconditioning ameliorated neuronal injury after middle cerebral artery occlusion by promoting neurogenesis

**DOI:** 10.1002/brb3.1804

**Published:** 2020-08-25

**Authors:** Lu Huang, Yaqi Wan, Zhancui Dang, Peng Yang, Quanyu Yang, Shizheng Wu

**Affiliations:** ^1^ Research Center for High Altitude Medicine Qinghai University Xining China; ^2^ Key Laboratory of Application and Foundation for High Altitude Medicine Research in Qinghai Province (Qinghai‐Utah Joint Research Key Lab for High Altitude Medicine) Xining China; ^3^ Qinghai Provincial People’s Hospital Xining China; ^4^ Qinghai University Medical College Xining China

**Keywords:** intermittent hypoxic precondition, middle cerebral artery occlusion, neural stem cell, neurogenesis, neuronal regeneration, subependymal ventricular zone

## Abstract

**Objectives:**

Sequelae of stroke were mainly caused by neuronal injury. Oxygen is a key factor affecting the microenvironment of neural stem cells (NSCs), and oxygen levels are used to promote NSC neurogenesis. In this study, effects of intermittent hypoxic preconditioning (HPC) on neurogenesis were investigated in a rat model of middle cerebral artery occlusion (MCAO).

**Methods:**

SD rats were used to establish the MCAO model. Nissl staining and Golgi staining were used to confirm the neuronal injury status in the MCAO model. Immunofluorescence, transmission electron microscopy, Western blot, and qPCR were used to observe the effects of HPC on neurogenesis. At the same time, the hypothesis that HPC could affect proliferation, apoptosis, differentiation, and migration of NSC was verified *in vitro*.

**Results:**

Hypoxic preconditioning significantly ameliorated the neuronal injury induced by MCAO. Compared with MCAO group, the dendrites, Edu^+^/SOX2^+^, Edu^+^/DCX^+^, Edu^+^/NeuN^+^, Edu^+^/GFAP^+^, and Edu^+^/Tubulin^+^ positive cells in the HPC + MCAO group exhibited significantly difference. Similarly, axonal and other neuronal injuries in the HPC + MCAO group were also ameliorated. In the in vitro experiments, mild HPC significantly enhanced the viability of NSCs, promoted the migration of differentiated cells, and reduced apoptosis.

**Conclusions:**

Our results showed that HPC significantly promotes neurogenesis after MCAO and ameliorates neuronal injury.

## Graphical abstract

1. HPC alleviated the nerve injury of MCAO; 2. HPC promoted neurogenesis; 3. HPC affects proliferation and apoptosis of NSCs.

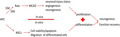

## 1 INTRODUCTION

Neuronal death and neurologic deficits are the major causes of disability in patients following cerebral infarction. Acute/programmed necrosis, autophagy, and apoptosis of brain neurons result from complex biochemical events triggered by stroke. Current treatments for cerebral infarction are limited; therefore, the development of effective treatment strategies would be of great value. At present, delivering thrombolysis in a timely fashion is the only feasible and effective means of rescuing the remaining neurons in patients after the onset of cerebral infarction, and tissue plasminogen activator is the only thrombolytic agent approved by the US Food and Drug Administration for use in clinical settings. The failure of regeneration, functional recovery of injured axons, and poor regeneration capacity of mature neurons are the primary causes of permanent disability following injury in the central nervous system (CNS) (Cho et al., 2015). Therefore, ensuring the neuroprotection and functional recovery of neurons following brain injury is challenging but essential.

Previous study has showed that the inhibition of serine racemase exerted neuroprotective effects after stroke and also improved aberration of cerebral blood flow (Watanabe et al., 2016). Distal ischemic preconditioning mediated neuroprotection following stroke by altering peripheral immune responses (Liu et al., 2016). Furthermore, ubiquitination of NCX3 has also become a novel target for enhancing neuroprotection induced by ischemic preconditioning (Cuomo et al., 2016). Likewise, molecular chaperones played a key role in neuroprotection in hypoxic‐ischemic encephalopathy (Hua, Ju, Jin, Sun, & Zhao, 2017). Neuroprotection following brain injury was primarily achieved through altering the pathways that mediate neuronal apoptosis, such as the mitochondrial pathway and exogenous Fas receptors, as well as the pathways induced by abnormalities in the endoplasmic‐reticulum calcium homeostasis and endoplasmic‐reticulum stress (Secondo et al., 2019).

Stem cells in the CNS are capable of self‐renewal and differentiation into neurons, astrocytes, and oligodendrocytes. Treatment of cerebral infarction with exogenous stem‐cell transplantation is gaining in popularity in current research. Studies have found that mesenchymal stem‐cell transplantation can repair neuronal injury by regulating CXCL12/CXCR4 signaling with the beneficial effects mainly achieved through the replacement of injured cells (Chau et al., 2018; Hu et al., 2019), immunomodulation (Chen et al., 2018), and neurotrophic actions (Abati, Bresolin, Comi, & Corti, 2019). However, exogenously transplanted stem cells have low survival rates possibly due to immune rejection, acute inflammation, and lack of neurotrophic signals (Wang et al., 2019).

Cerebral ischemic/hypoxic preconditioning (I/HPC) exerted endogenous protection enabling the brain to tolerate persistent ischemia/hypoxia. I/HPC could provide in‐depth protection, rendering it a potentially attractive treatment method (Gao et al., 2006). The role of oxygen in NSC proliferation and differentiation has been recently investigated. Hypoxic conditions promote the survival and proliferation of NSCs in the ganglionic eminence of mice. Hypoxia maintains the self‐renewal state of NSCs and affects their differentiation fate. It has been shown that hypoxia promoted neuronal NSC differentiation (Yuan, Guan, Ma, & Du, 2015), and the HPC of transplanted stem cells increases their capabilities to promote neuronal regeneration and functional recovery (Hu et al., 2019).

Therefore, in the present study, the mechanisms and effects of HPC on neurogenesis after MCAO based on the neuronal regenerating, immunomodulatory, and neurotrophic effects of NSCs were investigated, which lays the foundation for the therapy of the neurologic diseases in the future study.

## 2 EXPERIMENTAL PROCEDURES

### 2.1 Hypoxic preconditioning and MCAO

This experiment was performed under the supervision of the Institutional Animal Care and Use Committee of Qinghai University. The experimental procedures fully complied with animal ethics and protection guidelines. Adult male Sprague‐Dawley rats (280–300 g) were purchased from the Charles River Animal Center (Beijing, China). Rats were housed in a controlled environment with a 12 hr light–dark cycle and were randomly divided into four groups, including sham, HPC, HPC + MCAO, and MCAO groups (*n* = 8). The procedures for HPC and MCAO induction wereas previously described (Huang, Wu, Li, Dang, & Wu, 2019). Simply, Oxygen pressure and barometric pressure were, respectively, adjusted as 42 mmHg and 0.53 × 10^5^ Pa in a specific controlled environment, forming hypoxic environment. The conditions for HPC were performed for 3 hr every day at the same time (Yuan et al., 2015); the duration for HPC in this study was 10 and 20 days based on a single cycle of neurogenesis of 28 days (Khuu et al., 2019). Rats were anesthetized and established MCAO models after hypoxic preconditioning. The common carotid artery carefully isolated from midline incision in the neck. The filament was inserted after proximal ligation and distal end clamping of carotid artery and then ligated the suture. The experimental analysis was primarily performed on the subgranular zone (SGZ) tissue after 20 days of hypoxic pretreatment, including the marginal zone of the infarct and the ischemic region.

### 2.2 Nissl staining

Nissl staining was used to examine the status of neuronal injury. 4% paraformaldehyde was used to fix frozen sections, and the section was washed with distilled water after fixing 20 min. The sections were then stained with Nissl staining solution (Beyotime, C0117) for 10 min. Subsequently, the sections were washed twice with 70% ethanol, and the changes in the Nissl bodies were observed under a microscope.

### 2.3 Golgi‐Cox impregnation

The FD Rapid GolgiStain Kit (MKbio, Shanghai, China) was used to observe the microchanges in the morphology of dendritic spines and dendrites in the brain. The experimental procedures were performed according to the manufacturer's protocol.

### 2.4 Double immunostaining of immunofluorescence

Three nonserial sections were randomly selected from the marginal zone of the infarct for observation. Double immunofluorescent staining was performed on the tissues for neurogenesis analysis. The number and area of positive cells (DCX^+^/Edu^+^, SOX2^+^/Edu^+^, Tubulin^+^/Edu^+^, GFAP^+^/Edu^+^, and NeuN^+^/Edu^+^) in the SGZ were calculated for each image. The captured images were observed under a fluorescence microscope.

### 2.5 Western blot analysis

Tissues were lysed by ultrasonication, and protein concentration was determined using the bicinchoninic acid assay. An equal amount of total protein from each section was separated by polyacrylamide gel electrophoresis and transferred to a polyvinylidene fluoride membrane. Next, the membrane was blocked with 5% bovine serum albumin and then incubated with the special primary antibody and HRP‐labeled secondary antibody, respectively (Abcam, Cambridge, MA; ab205718). The primary antibodies were as follows: anti‐Doublecorin (Abcam, ab18723), anti‐NeuN (Abcam, ab177487), anti‐PCNA (Abcam, ab92552), and anti‐SOX 2 (Abcam, ab97959). Protein bands were obtained using enhanced chemiluminescence assay. Gray values were analyzed using image processing and analysis programs.

### 2.6 qRT‐PCR

The frontal cortex and the entire hippocampus were collected (Bhuvanendran, Kumari, Othman, & Shaikh, 2018). RNA was extracted using TRIzol and reverse‐transcribed into cDNA using the QuantiNova Reverse Transcription Kit (Qiagen, Hilden, Germany). The differential expression of each gene was analyzed following polymerase chain reaction (PCR) using the QuantiNova SYBR Green PCR Kit (Qiagen). The relative expression was defined as *F* = 2^−ΔΔct^. The primers were listed in Table 1.

**TABLE 1:** Primer sequences

Genes	Forward	Reverse
DCX	GTTTCTACCGCAATGGGGACC	CCAGTTGGGATTGACATTCTTGG
Fox3	GGGTTTTGGGTTTGTAACTTTTGAA	AGACTGCTCCTACCACAGGGTTTAG
β‐III‐Tubulin	TGAGGCCTCCTCTCACAAGT	TGTATAGTGCCCTTTGGCCC
HIF‐1ɑ	GCTGCCTCTTCGACAAGCTT	GCGTGGAGCTAGCAGAGTCA
MAP 2	ACCTTCCTCCATCCTCCCTC	AGTAGGTGTTGAGGTGCCGC
GAP 43	GGCTCTGCTACTACCGATGC	TTGGAGGACGGCGAGTTAT
EPO	GGGGGTGCCCGAACG	GGCCCCCAGAATATCACTGC
GLUT 1	TCTGGCATCAACGCTGTCTTC	CGATACCGGAGCCAATGGT
VEGF	CACAGCAGATGTGAATGCAG	TTTACACGTCTGCGGATCTT
Caspase 3	GGCAATCTGTACCTCTGCTTG	CGAGATGTCATTCCAGTGCT
Bcl‐2	AACCCCAGCGACTCTTTTATG	GGCAATCTGTACCTCTGCTTG
Bax	TCGAGGACGACTTCAACTATGG	ACAGCAAAATTAAGGCAGGACTC
GAPDH	TGTGGGCATCAATGGATTTGG	ACACCATGTATTCCGGGTCAAT

### 2.7 Transmission electron microscope

The injured brain tissue was immersed in chilled 4% glutaraldehyde and 1% acetic acid for fixation, followed by washes with phosphate‐buffered saline (PBS) and dehydration in an acetone gradient. The tissue was embedded in epoxy resin and sectioned. After negative staining with lead citrate, the sections were observed under a transmission electron microscope. The morphological observations in this experiment were conducted by three researchers who were experienced in the ultrastructure of the nervous system.

### 2.8 NSCs culture

Neural stem cells were isolated as previously described (Bernstock et al., 2019; Chiasson, Tropepe, Morshead, & van der Kooy, 1999; Morshead et al., 1994). The cells were cultured in Neurobasal medium. The growth medium comprised the DMEM‐F12 medium (Invitrogen Corp., Carlsbad, CA), 20 ng/ml of epidermal growth factor (R&D Systems, Minneapolis, MN), 10 ng/ml of basic fibroblast growth factor (R&D Systems), and 2% B27 (Thermo Fisher Scientific, Waltham, MA). The medium also contained 1% penicillin/streptomycin. The culture conditions were 37°C, 5% CO_2_, and 95% humidity. Cells of three passages or fewer were used in this experiment. Single‐cell suspension of NSCs was prepared, and the NSCs were identified using immunofluorescence. The anti‐Nestin antibody was used for labeling.

### 2.9 Oxygen glucose deprivation

The single‐cell suspension of NSCs was prepared by trypsinization. The cells were seeded into six‐well plates at a density of 1 × 10^5^ cells/ml. The culture medium was replaced with a glucose‐free medium, and the cells were incubated in a hypoxic environment. The condition of incubator was controlled as followed: 1% O_2_, 5% CO_2_, 94% N_2_, 37°C. The cells were subjected to treatment of oxygen glucose deprivation at different periods (3, 5, 8, and 10 hr) (Wang, Yang, & Wang, 2015).

### 2.10 Flow cytometry

Single‐cell suspensions were prepared from cells with different treatment times. The cells were centrifuged and washed with PBS, and 1.5 ml of chilled 70% ethanol was added. The cells were thoroughly mixed and placed at 4°C overnight. The next day, the cells were centrifuged, after which, propidium iodide (PI) was added to the cells in each group and incubated in the dark for 30 min. Flow cytometry was used to measure apoptosis.

### 2.11 Cell viability

NSCs were seeded into 96‐well plates. After incubation for 24 hr, the cells were subjected to oxygen and glucose deprivation for different periods of time. Subsequently, 10 μl of CCK‐8 solution was added, and the optical density value at 450 nm was measured after 4 hr of incubation to calculate cell viability.

### 2.12 Boyden chamber migration assay

The cell migration assay was mainly conducted with reference to a previous publication (Cui et al., 2019). Briefly, NSCs were incubated in a hypoxic and glucose‐free environment for 3, 5, 8, and 10 hr. Next, the cells were cultured in a differentiation medium for 4 days to measure the migration of the differentiated cells with different treatment times. The cells were seeded in the upper compartments of the Boyden chamber at a density of 1 × 10^5^ and incubated at 37°C for 24 hr. And then, the cells were treated with 4% paraformaldehyde and hematoxylin and eosin. The experiment was repeated thrice, and the number of migrated cells was averaged. Stromal cell‐derived factor 1 was used as a chemotactic factor.

### 2.13 Statistical analysis

SPSS.20 (IBM Corp., Armonk, NY) was used for the statistical analyses of the experimental data. All data are indicated as mean and standard error. One‐way analysis of variance was used for multi‐group comparisons. Student’s *t* test was used for between‐group comparisons. *p* < .05 was considered statistically significant. The GraphPad Prism software (GraphPad, La Jolla, CA) was used to plot the data.

## 3 RESULTS

### 3.1 Effects of HPC on neuronal injury after MCAO

**FIGURE 1:**
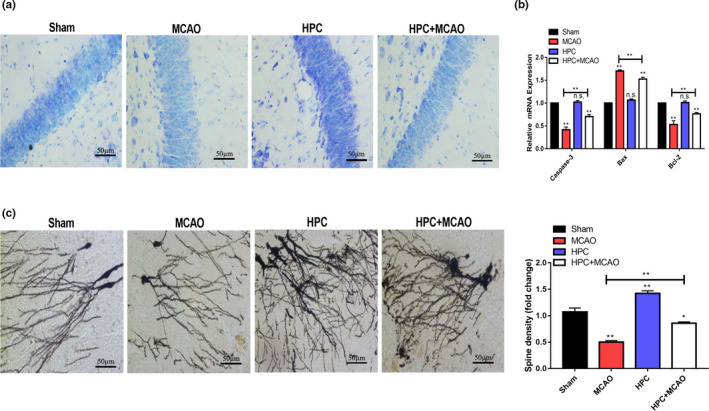
HPC alleviated the nerve injury of MCAO. (a) Nissl staining; (b) the changes of caspase 3, Bax and Bcl 2 expression in mRNA levels; (c) Golgi‐Cox impregnation (*p* < .05 was considered significant; * mean *p* < .05; **mean *p* < .01; n.s. mean *p* > .05)

As shown in Figure 1a, Nissl bodies in the neurons were stained purple–blue in the cytoplasm and light blue in the nuclei in all rat groups. Compared with the sham group, Nissl bodies significantly increased in the HPC group, whereas decreased in the MCAO group. These above results showed that neuronal injury was more severe in the MCAO group, whereas the HPC group exhibited active metabolic function and resulted in particularly abundant Nissl bodies. The Nissl staining of the HPC + MCAO group was of intermediate intensity. Short‐term preconditioning with intermittent hypoxia ameliorated cell apoptosis induced by MCAO to a certain extent. The expression level of Bax in the HPC + MCAO group was significantly lower, while the results of Bcl‐2 and caspase‐3 were reversed compared with the MCAO group (Figure 1b).

The results of Golgi staining were presented in Figure 1c. The loss and reduced density of dendritic spines were evident in the MCAO group, the dendrites were degenerated, and the dendritic trunks were fragmented. Varicosities appeared along with the dendritic spines, and the structures of the dendritic spines were destabilized or lost through degeneration in MCAO group. Compared to the MCAO group, the HPC and HPC+MCAO groups exhibited increased numbers of dendrites, and the normal morphology and structure of dendritic spines were maintained. The synapses were thickened and elongated, and the distribution of dendritic spines was denser.

### 3.2 Effects of HPC on neurogenesis after MCAO

**FIGURE 2:**
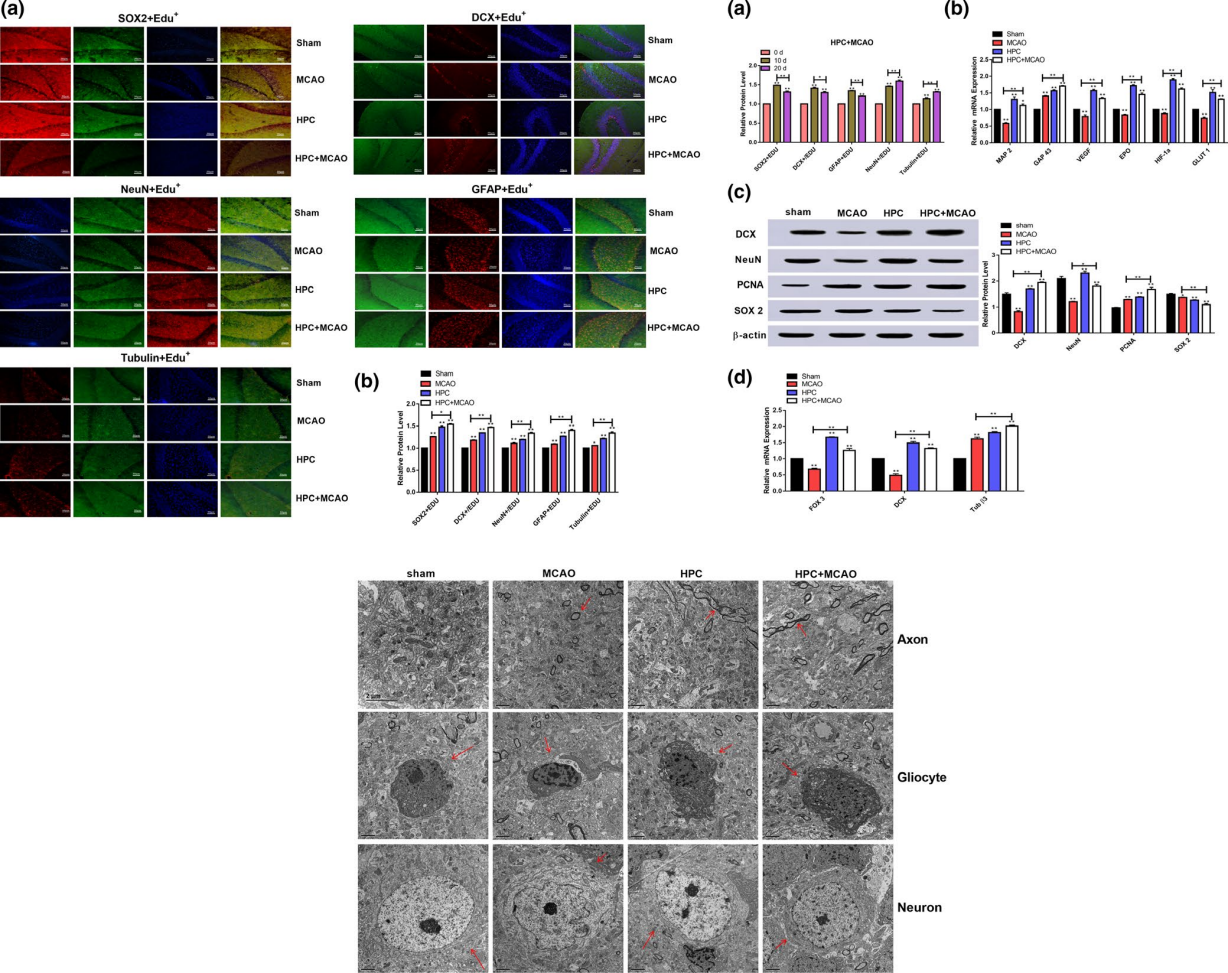
HPC promoted neurogenesis. (a) Neurogenesis was determined by fluorescent double labeling; (a: Edu^+^/SOX2^+^, Edu^+^/DCX^+^, Edu^+^/NeuN^+^, Edu^+^/GFAP^+^, and Edu^+^/Tubulin^+^; b: Statistical analysis). (b) The expression of neurogenesis‐related factor (a: The expression difference between 10 and 20d of related factor; b: RT‐PCR for MAP2, GAP43, VEGF, EPO, and HIF‐1ɑ; c: Western blot for DCX, NeuN, PCNA, and SOX2; d: The expression of FOX3, DCX, and ß‐III‐Tubulin in hippocampus). (c) The effect of HPC on ultrastructure of Axon, Gliocyte, and Neuron (*p* < .05 was considered significant; * mean *p* < .05; **mean *p* < .01; n.s. mean *p* > .05)

The study has reported that transient hypoxia did not result in brain apoptosis but led to an increase in cell density in the hippocampus with subsequent substantial neurogenesis (Pourié et al., 2006). As shown in Figure 2a, the results of double fluorescent labeling with Edu^+^ revealed that the expression levels of SOX2, GFAP, DCX, Tubulin, and NeuN were significantly higher in the MCAO, HPC, and HPC+MCAO groups than in the sham group, which indicated that the rat brains in these groups exhibited a higher degree of plasticity, and newborn cells were found in the SVZ and subgranular zone (SGZ) of the dentate gyrus (DG). More importantly, the HPC+MCAO group exhibited a high percentage of SOX2^+^/Edu^+^ cells. Compared with the MCAO group, the percentages of Tubulin^+^/Edu^+^ cells, DCX^+^/Edu^+^ cells, NeuN^+^/EDU^+^ cells, and GFAP^+^/Edu^+^ cells were higher in the HPC+MCAO group. These results suggested that neuronal migration and differentiation occurred 3 weeks after injury, and generally relied on the NSC microenvironment (Pourié et al., 2006).

To validate the processes of neurogenesis, the changes in neurogenesis with 10 days (data not shown) and 20 days of HPC were compared. As shown in Figure 2b, the expression levels of SOX2^+^/Edu^+^, DCX^+^/Edu^+^, and GFAP^+^/Edu^+^ cells were significantly higher in the 10‐day HPC group than in the 20‐day HPC group, whereas the expression levels of Tubulin^+^/Edu^+^ and NeuN^+^/Edu^+^ cells were significantly higher in the 20‐day HPC group. This result indicated that 10 days of HPC primarily promoted the formation of glial scars at the injury site, while 20 days of HPC promoted the differentiation of NSCs into functional neurons. The mRNAs levels of FOX3, DCX, and β‐III‐Tubulin in the hippocampal cortex of the HPC+MCAO group were significantly higher than those in the MCAO group. Similarly, the expression levels of DCX, NeuN, and PCNA proteins in the HPC+MCAO group were higher than those in the MCAO group, while the expression of SOX2 showed a downward trend after HPC.

The numbers of glial cells, neurons, and synapses decreased in the MCAO group (Figure 2c). Following HPC, there was a trend of restoration of these neurons, synapses, and glial cells. The neurons in the sham group displayed intact nuclei, abundant organelles, smooth‐surface endoplasmic reticulum, proper mitochondrial morphology, continuous membrane, and normal structure under a transmission electron microscope. Conversely, the neurons in the MCAO group were necrotic, morphologically damaged, with damaged nuclear membranes, unclear or disappeared cell boundaries, dissolved cytoplasm, swollen and dissolved mitochondria, and a large number of lysosomes and autophagosomes having appeared in the cell bodies. In the HPC+MCAO group, the formation of autophagosomes was reduced and the injuries to the neuronal cell bodies were attenuated. These findings indicated that HPC could protect the rat brain from MCAO‐induced neuronal apoptosis and ameliorate pathological damages to the microfiber and superfiber structures of the neurons, thereby exerting neuroprotective effects.

### 3.3 Effects of HPC on NSC proliferation and differentiation in vitro

**FIGURE 3:**
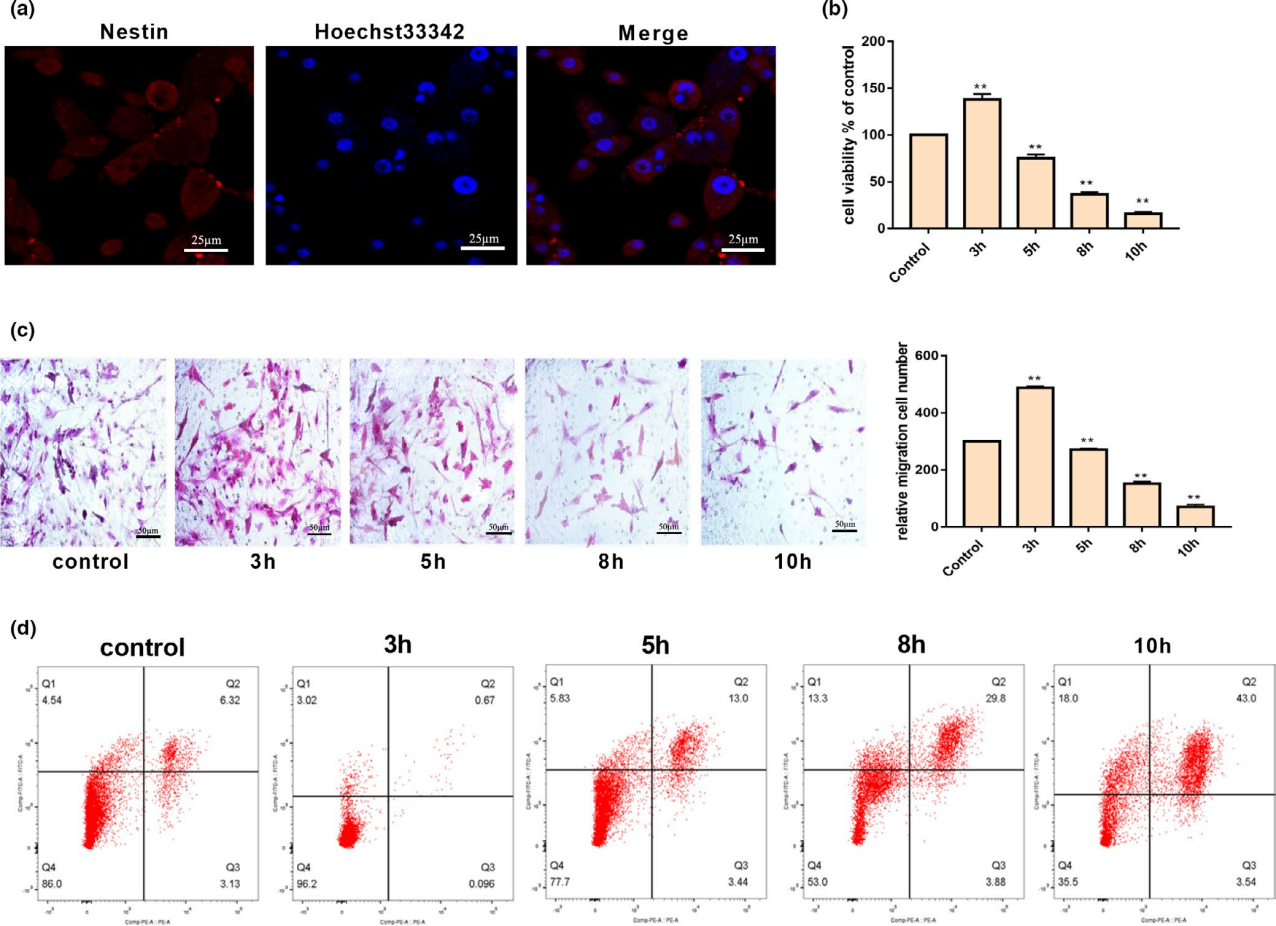
Effect of HPC on proliferation and apoptosis of NSCs. (a) The identification of NSCs; (b) HPC affect the cell viability; (c) effect of HPC on migration ability of differentiated NSCs; and (d) HPC affect cell apoptosis (*p* < .05 was considered significant; * mean *p* < .05; **mean *p* < .01; n.s. mean *p* > .05)

Indirect immunofluorescence labeling of Nestin was performed to identify the isolated NSCs. The expression of Nestin was shown in Figure 3a. Three hours of intermittent hypoxic treatment significantly promoted the cell viability of NSCs, while the cell viability gradually decreased after 3 hr treatment (Figure 3b), indicating that long‐term hypoxia was not beneficial to NSCs. A similar 3‐hr hypoxic treatment led to a marked enhancement in the migration ability of the differentiated NSCs (Figure 3c), while apoptosis decreased during NSC differentiation (Figure 3d). The migration ability of the differentiated cells decreased and NSC apoptosis increased with prolongation of the HPC time.

## 4 DISCUSSIONS

In our study, we examined the effects and mechanisms of HPC on neurogenesis after MCAO and found that HPC could dramatically ameliorate MCAO‐induced neuronal injury. Endogenous neurogenesis primarily occurs in the SVZ and SGZ of the DG, which are the regenerative niches of neural progenitor cells. Following injury, mature neurons in the SVZ migrated from the rostral migratory stream to the injured site, which constituted a self‐repair process after CNS injury (Chau et al., 2018). The newborn differentiated SGZ neurons can then participate in the local neuronal network of the hippocampus to regulate learning and memory. The abilities of endogenous NSCs to differentiate and migrate played an important role in the recovery of neuronal function after injury.

Hypoxia inhibits the activation of caspase 3, promotes the expression of HIF‐1ɑ, and enhances the expression of brain‐derived neurotrophic factor and vascular endothelial growth factor (VEGF), thereby promoting angiogenesis and neurogenesis, which reduce neuronal death and ameliorate neuronal function after MCAO (Chen et al., 2017). Hypoxia stimulation increased the stemness of somatic cells through reprogramming (Nakagomi, Nakano‐Doi, Narita, & Matsuyama, 2015), altered the microenvironment of neural progenitor cells, and stimulates cell proliferation. MCAO inhibits synaptic plasticity and reduces dendritic spine and synaptic densities, eventually leading to neuronal injury. HPC can stimulate glia‐mediated synapse formation and reduce neuronal injury after MCAO, thereby reducing the cerebral infarct volume. Studies showed that HPC could increase c‐Fos expression in newborn hippocampal cells (Tsai, Yang, Wang, & Wang, 2011), which in turn affected synaptic plasticity and hippocampal adult neurogenesis (Khuu et al., 2019).

The neurovascular niche can affect functional recovery after stroke, and it is essential for nerve growth factors, neurotrophic factors, and oxygen transport. VEGF can promote neurogenesis and the endogenous migration of neurons in the SGV and SVZ (Wang et al., 2008). Many studies have shown that neurogenesis and angiogenesis are coordinated and occur simultaneously in the SVZ. Endothelial cells promote the differentiation of NSCs into neurons, while the NSCs promote the formation of vascular structures (Hongjin, Han, Baoxiang, Shiqi, & Xiaoyu, 2019). Both endogenous NSCs and exogenous, transplanted NSCs can sense and migrate to the lesion site along the chemokine concentration gradient, where they differentiate into specific cells, release neurotrophic factors, or perform immune modulation to regulate tissue homeostasis in the lesion site. Local tissue releases VEGF and erythropoietin (EPO) under HIF‐1ɑ induction, which can promote the self‐renewal of NSCs.

This is consistent with our findings, which showed that HPC enriches the Nissl bodies and increases dendritic spine density, and the dendritic structures tend to become more functional. The expression of MAP2 and Tuj1 also increases, and a neuronal network with axons is established for neurotransmitter signal transduction. Meanwhile, HPC significantly increases the number of newborn cells in the hippocampus, as well as the expression of markers for newborn neuronal precursors and mature differentiated neurons, such as GAP43, DCX, NeuN, and SOX2, which are significantly elevated. These findings confirm the occurrence of neurogenesis and show that the process of neurogenesis involves changes from proliferation to differentiation. Furthermore, under hypoxic conditions, the high expression of HIF‐1ɑ increases the expression of angiogenesis‐related factors, such as VEGF, EPO, and GLUT, which coordinate and interact with neurogenesis.

## 5 CONCLUSION

The treatment of HPC could relieve the nerve injury of MCAO in morphology and molecular levels. Meanwhile, the therapy effect of appropriate HPC treatment was realized through enhanced the viability of NSCs cell and promoted the migration ability of differentiation cells.

## CONFLICT OF INTEREST

None.

## AUTHOR CONTRIBUTIONS

Lu‐huang wrote the writing of manuscript and performed the experiments; Ya‐qi wan and Zhan‐cui Dang analyzed the data; Peng‐yang and Quan‐yu Yang supervised the research; Shi‐zheng Wu designed this study and responsible for the submitting manuscript.

### Peer Review

The peer review history for this article is available at https://publons.com/publon/10.1002/brb3.1804.

## Data Availability

Data are available on request from corresponding author.
